# Association between Platescapes, Foodscapes, and Meal Energy Intake in Government Employees from Muar, Johor, Malaysia

**DOI:** 10.3390/nu10070819

**Published:** 2018-06-25

**Authors:** Ying Jye Lim, Rosita Jamaluddin, Ying Ting Er

**Affiliations:** Department of Nutrition and Dietetics, Faculty of Medicine and Health Sciences, Universiti Putra Malaysia, Serdang 43400, Malaysia; yingjye806@gmail.com (Y.J.L.); yingting0825@gmail.com (Y.T.E.)

**Keywords:** foodscapes, platescapes, energy intake, meal energy, quasi-experiment

## Abstract

A microscale built environment was the focus in this cross-sectional study which aimed to investigate the associations between platescapes, foodscapes, and meal energy intake among subjects. A total of 133 subjects (54 male, 79 female) with mean age 36.8 ± 7.3 years completed a self-administered questionnaire on sociodemographic characteristics, platescapes, and foodscape preferences. For platescapes, a plate mapping method was used, where subjects were required to place various sizes of food models on two different sized plates (23 cm and 28 cm) based on their preferences. For foodscape preferences, subjects were given a 23-cm plate and various food models differentiated by shapes and colours. Then, 24-h daily recalls (for one weekday and one weekend day) were obtained using interviews. Significant differences were observed in meal energy intake (*p* < 0.05) between males (1741 ± 339 kcal) and females (1625 ± 247 kcal) and also between age groups (*p* < 0.05). There was a significant difference (*p* < 0.0001) in terms of subjects’ meal energy intake when comparing 23-cm plates (419 ± 124 kcal) and 28-cm plates (561 ± 143 kcal). The bigger plate (28 cm) (*p* < 0.01) was significantly associated with subjects’ meal energy intakes, but this was not so for the 23-cm plate. There were significant differences in subjects’ meal energy when comparing white rice and multicoloured rice (*p* < 0.0001), unicoloured and multicoloured proteins (*p* < 0.0001), and unicoloured and multicoloured vegetables (*p* < 0.0001). There was a significant difference found between round- and cube-shaped proteins (*p* < 0.05). The colours of rice (*p* < 0.01), protein (*p* < 0.05), and vegetables (*p* < 0.05) were significantly associated with subjects’ meal energy. Only the shape of carrots in vegetables (*p* = 0.01) was significantly associated with subjects’ meal energy. Subconsciously, platescapes and foodscapes affect an individual’s energy intake, and thus these elements should be considered in assessing one’s dietary consumption.

## 1. Introduction

In recent years, new terminology has appeared in combination with the suffix ‘-scape’, including the terms “platescapes”, “foodscapes”, “kitchenscapes”, and “tablescapes”. These are considered elements of the microscale built environment. In this context, elements such as platescapes and foodscapes may also influence adult energy intake, and this may be reflected in body weight. The term “platescape” refers to the appearance of the container from which the food is consumed, and includes characteristics such as plate shape, plate size, bowl shape, bowl size, utensils, and also glass shape. The term “foodscapes” refers to the view and/or appearance of an edible item that will be consumed, and involves elements such as sizes, shapes, textures, colours, and divisions or distinctions apparent on the surface of the food [1]. The influence of microscale environments on energy intake can be explained from the perspective of food choices that are rooted in the built environment [2,3] such as dining environment and settings, utensils and cutlery used, appearance of food of different colours, and cutting shape and size, as well as textures [1]. The built environments of food could affect decisions on types and amounts of food to be consumed, and eventually the energy intake. Microlevel built environments, including platescapes (plate size) and foodscapes (colour and shape) are the focus of this study. Plate mapping is a new method of measuring meal and portion size, and the results will be interpreted in order to determine whether plate size influences energy intake. Different plate sizes [4,5], food portion sizes [6,7], and colours [8] may influence the total energy intake as the amount of food consumed by individuals will be different.

Dietary intake is measured by using dietary recall, household measurement, food frequency questionnaires (FFQs), and food preparation methods. However, there is a lack of studies reporting on nutrient intake of adults in Malaysia with respect to the influence of the built environment on energy intake. Few studies focusing on the influence of perception of platescapes as well as foodscapes on energy intake, particularly among Malaysian adults, have been reported. Thus, the objective of this study is to study the influence of perception of platescapes and foodscapes on energy intake among government employees in Muar, Johor, Malaysia.

## 2. Materials and Methods

### 2.1. Study Design

This was a quasi-experimental study design conducted to assess the influence of platescapes and foodscapes on energy intake, and subjects were recruited from a government office in Muar, Johor. The subjects were selected at only one point in time to participate in the study. Thus, the data and information collected reflect the current situation and opinions of the subjects at only one point in time.

### 2.2. Data Collection

Prior to the commencement of the study, ethical approval to conduct the study was obtained from the Ethics Committee for Research Involving Human Subjects (JKEUPM) from Universiti Putra Malaysia. Permissions were also obtained from the government office headquarter in Putrajaya and the Muar office branch for data collection.

Subjects aged 18 to 60 years were invited to participate and were given an information sheet explaining the purpose of the study. Written informed consent to participate was obtained prior to administration of the questionnaire. Two-day face-to-face interviews were conducted to obtain the information on dietary intake by the researcher. Following the completion of the questionnaire, data on plate mapping and foodscape preference were obtained from subjects, with food models provided.

### 2.3. Sample Size

Sample size was estimated using the equation of Aday and Cornelius (2006). The mean and standard deviation from a previous study were used for sample size calculation for the quasi-experimental study. On including an α error of 0.05 and β error of 0.20, a total of 109 subjects were required. Another 20% was added to allow for the drop-off rate, and thus the total required sample size in this study was set at 131 subjects.

### 2.4. Measures

#### 2.4.1. Socio-Demography

A socio-demographical information questionnaire was self-administered by the subjects. Information on date of birth, age, sex, ethnicity, highest educational level, monthly personal income, monthly household income, and marital status of the subjects was obtained.

#### 2.4.2. Plate Mapping

**Figure 1:**
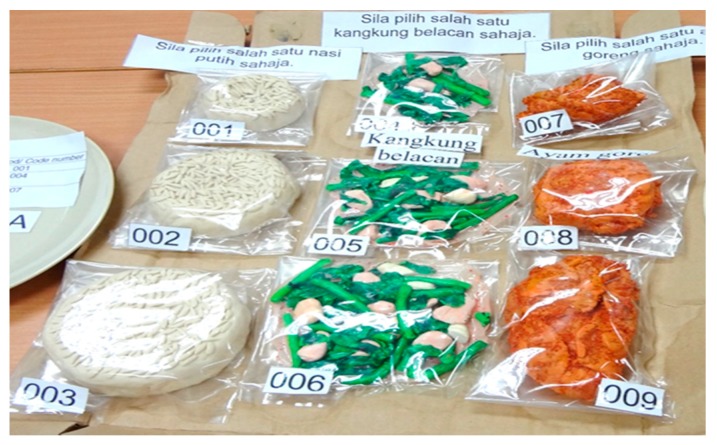
Food models for carbohydrates, vegetables, and protein, with different portion sizes.

Two different sizes of plates (measuring 23 cm and 28 cm) were provided to each of the subjects. The plate sizes were determined based on a previous study to determine the influence of plate size on energy intake [9]. Next, the subjects were required to choose the various food models (Figure 1) provided for carbohydrates, vegetables, and protein based on their preferences. The portions chosen were then translated into the corresponding weight and energy content of the food. The energy content denoted by the food models was calculated based on the common recipes entered into Nutritionist Pro Diet Analysis software. The data was processed based on Malaysian Food Composition Database [10] using Nutritionist Pro Diet Analysis software. The total energy of each type of food as shown in Table 1 was added to show the amount of energy of the meal on the plate.

#### 2.4.3. Foodscapes Preferences

The two criteria investigated under foodscapes were colour and shape. Subjects were only given a 23-cm plate and instructed to choose various food models differentiated by colours and shapes based on their preferences. Then the portion chosen was translated into the corresponding weight and energy content of the food. The total energy of each food was then added to show the amount of energy content of the meal on the plate.

#### 2.4.4. Colour Criterion

**Figure 2:**
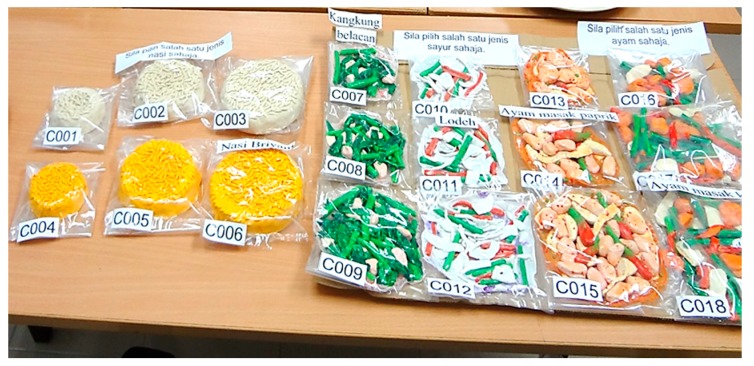
Food models for foodscapes preferences—colour criterion.

Food models of carbohydrate, protein, and vegetables were provided in three different amounts to subjects. Different-coloured carbohydrate-based foods were provided (white rice and flavoured rice (Briyani Rice). The protein-based foods included Paprika Chicken (unicoloured) and Turmeric Chicken (multicoloured). The vegetables were stir-fried water spinach (unicoloured) and *Lodeh* (multicoloured). The subjects were requested to choose one of these food models as shown in Figure 2. Table 2 displays the portion size of different colours of foods with weight and energy content.

#### 2.4.5. Shape Criterion

**Figure 3:**
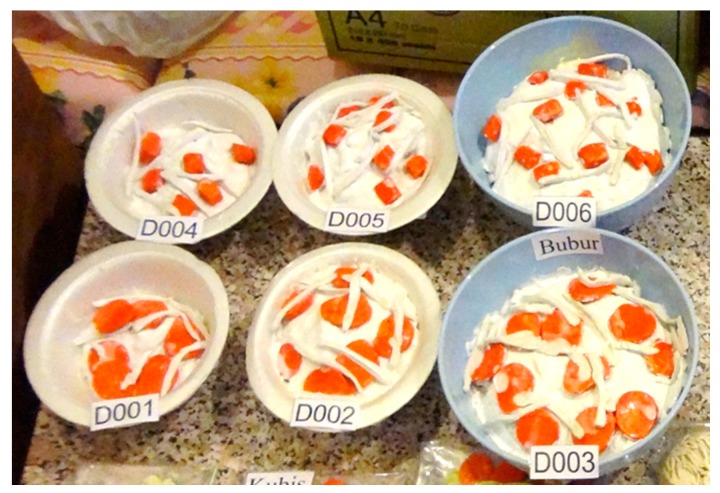
Food models for foodscape preferences—shape criterion (for carbohydrates).

**Figure 4:**
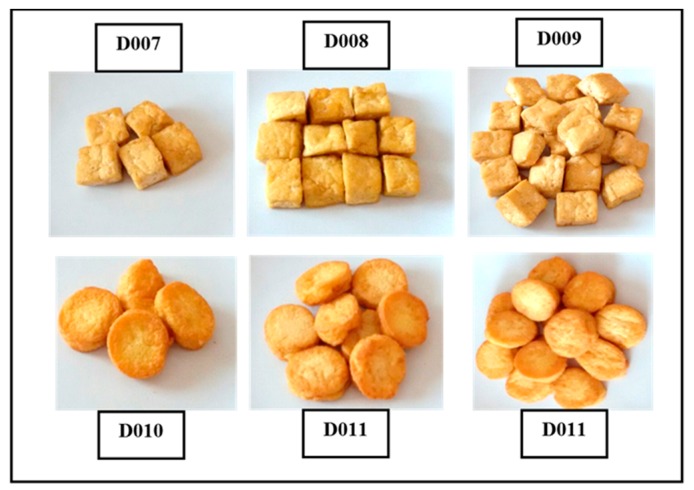
Food models for foodscapes preferences—shape criterion (for proteins).

**Figure 5:**
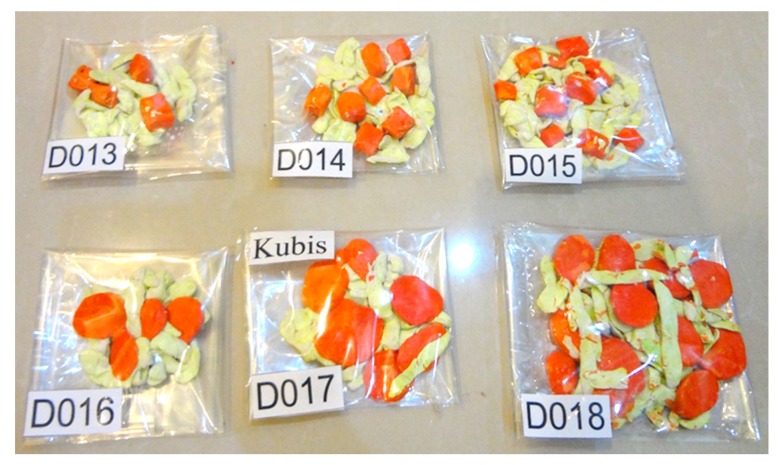
Food models for foodscapes preferences—shape criterion (for vegetables).

For the shape criterion, subjects were given food models in a cube shape or round shape in three different serving portions. For carbohydrates, rice porridge with shredded chicken was mixed with either cube-shaped or round-shaped carrot (Figure 3); protein-based foods were either fried round tofu or fried cubed tofu (Figure 4); and the vegetables were stir-fried cabbage with either cube-shaped or round-shaped carrot (Figure 5). The portion size of different shapes of foods with weight and energy content are presented in Table 3. The subjects were requested to choose one of these food models as shown in the figures.

#### 2.4.6. Energy Intake

In addition, the dietary intakes of the subjects were measured using 24-h dietary recall from two days (one weekday and one weekend day). Detailed descriptions of all foods and beverages including cooking methods and brand names of processed foods were recorded. The portion sizes of the food intakes of the subjects were estimated based on the standard household measurement tools [11]. Energy intake was analysed based on the Malaysian Food Composition Database [10].

### 2.5. Statistical Analyses

Data analysis was performed by using SPSS version 22.0 for Windows (SPSS Inc., Chicago, IL, USA). Continuous variables were presented as means and standard deviations, whereas categorical variables were presented as frequencies and percentages. Pearson correlation and Chi-square were used to determine the associations between built environment factors (platescapes and foodscapes) with meal energy. Furthermore, the *t*-test was used to compare the perception of platescapes and foodscapes. ANOVA was used to compare mean between sociodemographic factors and meal energy. The level of statistical significance was set at *p* < 0.05. Nutritionist Pro Diet Analysis version 2.4.1 was used to analyse the energy intake of the subjects based on 24-h diet recall.

## 3. Results

### 3.1. Subjects’ Characteristics

Out of 150 employees, a total of 133 subjects agreed to participate in this study and completed the self-administered questionnaire. The socio-demographic characteristics of the subjects are described in Table 4. As shown in the table, a greater number of female subjects participated (59.4%), and more than half of the subjects were in the age range of 30 to 39 years old. Mean age was 36.83 ± 7.26 years old. Most of the subjects were Malays (95.5%). As for the educational level, the majority of the subjects (75.2%) had completed secondary school. Around nine out of ten of the subjects (88.0%) were married. Moreover, were more than half of the subjects had monthly personal income (87.2%) and household income (56.4%) in the range between Malaysia Ringgit RM1001 and RM3000 (approximately between USD251 and USD752).

### 3.2. Energy Intake

The energy intake obtained through two-day 24-h dietary recalls (one weekday and one weekend day) was identified using nutrient composition analysis. The mean total energy intake was computed and the difference between sexes was determined. As displayed in Table 5, a significant difference in energy intake was found between male and female respondents (*p* < 0.05). Male respondents had slightly higher mean total energy intake (1741 ± 339 kcal) compared to female respondents (1625 ± 247 kcal). 

Table 6 presents the difference between age groups and total energy intake. Respondents in the age group of 30 to 39 years had the highest mean energy intake (1721 ± 263 kcal) whereas the lowest mean energy intake (1564 ± 355 kcal) was found among respondents who were over 40 years old. Statistically significant differences in energy intake were reported between age groups (*p* < 0.05). Table 7 shows the Turkey post hoc test conducted to identify the differences between age groups. There was a significant difference in total daily energy intake between the groups aged 30 to 39 years and 40 years or more (*p* = 0.017). However, there were no significant differences between the other age groups. It can be further concluded that adults aged 40 years or more consumed less energy as compared to those aged 30 to 39 years.

### 3.3. Differences and Relationship of Platescapes, Preferences with Food Portion Sizes and Meal Energy

The preferences of food portion size using a 23-cm plate and 28-cm plate are shown in Table 8. From the analysis, it was consistently shown that a bigger portion was chosen when a bigger plate was given. Most of the subjects chose two scoops of rice (57.9%) whereas there were only 7.5% of them chose three scoops of rice when a 23-cm plate was provided. However, when 28-cm plate was given, the majority of them chose two scoops of rice (44.4%), while the number of subjects who chose three scoops of rice increased to 39.8%. The increment in the number the subjects selecting three scoops of rice was 32.3% for a 28-cm plate as compared to a 23-cm plate, whereas a decrement was reported for subjects choosing one scoop of rice (18.8%) and two scoops of rice (13.5%). Significant differences were found in the number of subjects who chose one scoop of rice and three scoops of rice (*p* < 0.001) when different plate sizes were given.

The same process was applied to protein and vegetables. Subjects were more likely to choose a moderate-sized (56.4%) and large-sized portion of fried chicken (26.3%) when a 28-cm plate was provided as compared to a 23-cm plate. There were increments of 21.0% of large-sized fried chicken and 14.3% of moderate-sized fried chicken, with a decrement in the small-sized fried chicken portion (−35.3%) when a 28cm plate was given (*p* < 0.001).

For vegetables, the majority of the subjects chose half a scoop (48.9%), followed by one scoop (39.1%) when a 23-cm plate was provided. When a 28-cm plate was provided, most subjects chose one scoop of stir-fried water spinach (60.9%) followed by one and half scoops (29.3%). There was an increment in the proportion of subjects choosing one scoop (+21.8%) and one and half scoops of stir-fried water spinach (+17.3%) for the 28-cm plate as compared to the 23-cm plate, where there was a decrement in the proportion of subjects choosing a half scoop of stir-fried water spinach (−39.1%). Significant differences in the numbers of subjects who chose half a scoop, one scoop, and one and half scoops of stir-fried water spinach were reported (*p* < 0.001) between the two plate sizes.

Table 9 displays the comparison of the meal energy content based on plate size between genders. For carbohydrate, protein, and vegetable food, significant differences in meal energy were found (*p* < 0.001). This indicated that when subjects were given a bigger plate, they tended to take more scoops of rice as compared to when a smaller plate was given. Moreover, male subjects (23-cm plate: 181 kcal; 28-cm plate: 231 kcal) chose more calories for rice compared to female subjects (23-cm plate: 167 kcal; 28-cm plate: 219 kcal). There was an increment of 28% of meal energy for rice in male subjects and 31% in female when bigger plates were provided. In the long term, negative health consequences are postulated when a bigger plate is given due to the increase in total energy intake.

The energy content of fried chicken chosen by subjects in this study was significantly different according to plate sizes (*p* < 0.001). Furthermore, male subjects (23-cm plate: 210 kcal; 28-cm plate: 295 kcal; 40% energy increment) had a higher energy intake with respect to fried chicken as compared to female subjects (23-cm plate: 202 kcal; 28-cm plate: 273 kcal; 35% energy increment).

For stir-fried water spinach, the meal energy by the subjects in the study was significantly different based on plate sizes (*p* < 0.001), as subjects took more scoops of stir-fried water spinach when a bigger plate was given. Females were reported to have higher meal energy intake from stir-fried water spinach as compared to males. Furthermore, male subjects (39% energy increment) tended to have a greater increase in meal energy for stir-fried water spinach as compared to females (33% energy increment). This showed that when bigger plates were given, male subjects had a greater energy intake for stir-fried water spinach as compared to females. In the long term, a positive impact is hypothesized when bigger plates are given because the number of scoops of stir-fried water spinach taken by subjects increases.

Overall, higher meal energy intake was noted when a bigger plate was given among subjects. Both genders had greater total meal energy from a 28-cm plate as compared to the total meal energy from a 23-cm plate (*p* < 0.01) with a 33.9% energy increment when a bigger plate was given.

### 3.4. Differences and Associations of Foodscapes Preference (Colour and Shape) with Meal Energy

Table 10 and Table 11 display the differences between foodscapes preference and comparison of meal energy in accordance with colour criterion. For this part, only a 23-cm plate was given to the subjects, and most of them chose multicoloured foods rather than unicoloured foods. When the selection of the food between the colours was compared, it was found that more subjects selected multicoloured rice (+18.8%) and multicoloured protein food (+44.4%) as well as multicoloured vegetables (+30.8%). Significant differences were reported between unicoloured and multicoloured food (*p* < 0.05). As for the comparison of meal energy between unicoloured and multicoloured food (Table 11), there were increments in meal energy for multicoloured rice (+54%), multicoloured protein (33%), and multicoloured vegetables (159%) as compared with unicoloured foods (*p* < 0.001) due to the additional ingredients added which increased the energy value of the dishes.

The differences between foodscapes (shape) preference based on food type (Table 12) and comparison of meal energy (Table 13) were analysed and displayed. When the selection of the food between the shapes was compared, more subjects selected chicken porridge (+66.9%) and stir-fried cabbage with round carrot (+54.9%) rather than cubed carrot. Table 13 displays the comparison of meal energy between round- and cube-shaped food. From the analysis, it was revealed that fried cubed tofu contributed 22% more meal energy than fried round tofu (*p* < 0.05). However, there were no significant differences based on the shape of carrot in porridge and vegetables (*p* > 0.05) as chosen by subjects.

Furthermore, the associations between foodscapes (colour and shape) and total meal energy were determined and are displayed in Table 14. The analysis was conducted to identify the effect between the foodscape of colour (unicoloured and multicoloured) as well as foodscape of shape (round and cube shaped) on total meal energy among the respective subjects.

The cut-off point of meal energy was set as 1500 kcal, as the mode was 1549 kcal and the mean was 1670 kcal. Subjects who chose multicoloured rice were more likely to have higher total meal energy, with 68.5% of the subjects consuming more than 1500 kcal for multicoloured rice, whereas 61.0% consumed less than 1500 kcal when they chose unicoloured (white) rice (*p* < 0.05). When protein was added, 78.3% of subjects consumed more than 1500 kcal when selecting multicoloured protein, while 41.5% consumed less than 1500 kcal when unicoloured protein was selected. A significant association was reported between protein colour and meal energy (*p* < 0.05). For vegetables, a significant association between colour of vegetables and meal energy was also reported (*p* < 0.05). Subjects (56%) who added unicoloured vegetables onto the plate tended to consume less than 1500 kcal. In contrast, those who chose multicoloured vegetables (75.0%) tended to consume more than 1500 kcal.

A significant association was reported between the shape of carrots and subjects’ meal energy (*p* < 0.05). It can be concluded that there were more subjects who preferred round-shaped carrot compared to cube-shaped carrot. As for the shape criterion in porridge and protein foods, no significant differences were reported (*p* > 0.05).

## 4. Discussion

In this study, subjects who were provided with bigger plate size consistently tended to have greater meal energy values, choosing foods with larger portion sizes. This is consistent with Pratt et al. (2011) who showed that a slight increase in the dishware size will cause a significant increase in the amount of energy to be consumed [12]. This is due to the misperception of the need for larger portion sizes when given bigger plate sizes [13,14]. Furthermore, both men and women tended to consume more foods with bigger plate size, indicating that platescapes do have an impact on energy intake. However, this finding contradicted a previous study which found no significant differences between men and women in total food area or proportion of plate covered for large as well as small plates [15].

It was reported that for normal-sized dinnerware, people appear to visually anchor an around 70% fill level for dinnerware, and past research claimed that individuals will normally eat around 92% of food which they have served themselves [16,17]. The DelBoeuf illusion is defined as where an item looks smaller when surrounded by larger items [18,19], with foods looking larger when they are on a smaller plate. Thus, subjects tend to take more when a bigger plate is given to them and eventually this causes more energy consumed by the subjects. In addition, larger plates will cause people to portray their meals as being bigger, whereas smaller plates will cause people to portray their meals as smaller [9].

Furthermore, subjects in the current study took 34% more food when they were provided with 28-cm plate. The current finding is supported by a previous study where when college students were given 23-cm and 28-cm plates they drew 26% more food onto the bigger plate than the smaller one [9]. When a bigger bowl was received by the professionals, they served and consumed over 31% more ice cream compared to those who received a smaller bowl [7,18]. In addition, Chinese buffet guests who chose bigger plates were served 52% extra food and ate 45.1 percent more compared to those who chose a smaller plate.

On the contrary, there were studies which found no effect of the plate size on energy intake of an individual. One study mentioned that there was no obvious decrement in food intake among the overweight ladies even when a smaller plate was used during a buffet setting [20]. Another study showed that a reduction in food consumption at meals eaten in the laboratory did not occur when using a smaller plate [21].

In addition, the foodscape colour criterion determined in the current study found that subjects who chose multicoloured foods had higher meal energy compared with unicoloured food. This study reported the same results as in a previous study, where it was found that different-coloured foods led to greater food ingestion. When the number of food colours was increased from six colours to 24 colours, the amount of food consumed by the participants increased, with significant differences [8]. 

Multicoloured food offers limited signs about at what time to start or stop consuming [1]. This situation is further explained by one study in which even when the taste of each colour was the same, subjects who had received a bowl with ten colours consumed 43% more (91 versus 64 candies) within an hour compared to subjects who had received seven colours [6]. Furthermore, food intake and food choice are influenced by several external factors which are social and physical surroundings, presence of other people and sound, temperature, smell, colour, time, and distractions [22]. The results obtained from this study were consistent with a previous study which found that stripes, swirls, and segmented colours might be useful in order to choose where to consume from and the size as well as number of bites to take [1]. At the same time, the results of the current study were also in agreement with a study which found that when three flavours of yoghurt that varied in flavour, texture, and colour were given in succession, significantly more yoghurt (*p* < 0.01) was consumed by subjects than when only one flavour was given; even though the single flavour was the subjects’ favourite (*p* < 0.01) [23].

As for the foodscape shape criterion, subjects preferred round-shaped carrot for their porridge rather than cube-shaped carrot. The findings are in agreement with a previous study where it was concluded that sphere shape food looks smaller compared to a flattened square sheet food, so round food is more likely to be fully consumed compared to square-shaped food (χ^2^ = 6.88, *p* < 0.01) [24]. In addition, there was a significant enhancement (*p* < 0.025) in food consumption with an assortment of food shapes [23]. However, this finding contraindicated another study that found that the differences between the cross and triangle shapes were significant, but among the square and the circle shapes no statistically significant differences were found [25]. There are numerous inconsistent findings on comparative size perceptions of circles versus squares [24]. Research found that the same area of a circle was perceived to be smaller than a square [26]. Moreover, the opposite result was found in subsequent studies [27,28,29,30] and no difference between the comparative sizes perceptions of circles versus squares have been found by other studies [25,31].

## 5. Conclusions

In conclusion, there were increments in meal energy when bigger plates (28 cm ere) were given to subjects. As for the foodscape colour criterion, there was an increment in term of portion intake for multicoloured food. For the foodscapes shape criterion, more subjects chose round-shaped food compared to cube-shaped food. In the long term, a larger plate will lead to a negative impact as the increased number of scoops of rice and larger portions of fried chicken consumed increase the total energy intake. However, the increment in vegetable intake when bigger plates are given will have a positive impact. The present study provides a better understanding of the influence of the built environment on energy intake among adults, which may provide a better insight into developing a more effective strategy for promoting healthier food choices and food intake. This study provides baseline data for future research on the influence of built environment factors on dietary intake in the population.

## Figures and Tables

**Table 1:** Participants’ socio-demographic and anthropometric data at baseline.

Foods	Portion Size	Code Number	Weight (g)	Energy Content (kcal)
**Carbohydrate food** **(white rice, boiled)**	1 scoop	001	75	100
	2 scoops	002	150	200
	3 scoops	003	225	300
**Vegetable food** **(stir-fried water spinach)**	½ scoop	004	30	25
	1 scoop	005	60	50
	1½ scoops	006	90	75
**Protein food** **(fried chicken)**	1 small-sized	007	60	135
	1 moderate-sized	008	120	270
	1 big-sized	009	180	405

**Table 2:** Portion size of different colours of foods with corresponding weight and energy content.

Foods	Portion Size	Types of Food Weight (g)/Energy Content (kcal) [Code Number]	
**Carbohydrate food**		White rice, boiled	Briyani rice (Flavoured rice)
	1 scoop	75/100 (C001)	75/138 (C004)
	2 scoops	150/200 (C002)	150/276 (C005)
	3 scoops	225/300 (C003)	225/414 (C006)
**Vegetable food**		Stir-fried water spinach (Unicoloured)	*Lodeh* (Multicoloured)
	½ scoop	30/25 (C007)	40/65 (C010)
	1 scoop	60/50 (C008)	80/130 (C011)
	1½ scoops	90/75 (C009)	120/195 (C012)
**Protein food**		*Paprika chicken* (Unicoloured)	*Turmeric chicken* (Multicoloured)
	1 small-sized	70/70 (C013)	50/80 (C016)
	1 moderate-sized	140/140 (C014)	100/160 (C017)
	1 large-sized	210/210 (C015)	150/240 (C018)

**Table 3:** Portion size of different shapes of foods with corresponding weight and energy content.

Foods	Portion Size	Types of Food Weight (g)/Energy Content (kcal) [Code Number]	
**Carbohydrates (mixed porridge with shredded chicken and carrot)**		Cube-shaped	Round-shaped
	½ bowl	75/70 (D004)	75/70 (D001)
	1 bowl	150/140 (D005)	150/140 (D002)
	1 ½ bowls	225/210 (D006)	225/210 (D003)
**Proteins (fried tofu)**		Cube-shaped	Round-shaped
	½ scoop	45/70 (D007)	45/60 (D010)
	1 scoop	90/140 (D008)	90/120 (D011)
	1½ scoops	135/210 (D009)	135/180 (D012)
**Vegetables (stir-fried cabbage with carrot)**		Cube-shaped	Round-shaped
	½ scoop	30/25 (D013)	30/25 (D016)
	1 scoop	60/50 (D014)	60/50 (D017)
	1½ scoops	90/75 (D015)	90/75 (D018)

**Table 4:** Socio-demographic characteristics of subjects (*n* = 133).

Socio-Demographic Characteristics	Frequency (*n*)	Percentage (%)	Mean ± SD
Sex			
Male	54	40.6	
Female	79	59.4	
Age (years)			36.83 ± 7.26
20–29	2	18.8	
30–39	67	50.4	
40–49	33	24.8	
≥50	8	6.0	
Ethnicity			
Malay	127	95.5	
Chinese	2	1.5	
Indian	4	3.0	
Highest educational level			
Completed Form 5	100	75.2	
Completed Form 6/certificate/diploma	30	22.6	
Completed Bachelor’s degree	3	2.3	
Marital status			Mean age (years)
Single	11	8.3	35.27 ± 8.08
Married	117	88.0	36.98 ± 7.28
Divorced/widowed	5	3.8	36.60 ± 5.41
Monthly personal income			
RM1001–RM3000	116	87.2	
RM3001–RM5000	16	12.0	
>RM5000	1	0.8	
Monthly household income			
RM1001–RM3000	75	56.4	
RM3001–RM5000	45	33.8	
RM5001–RM7000	10	7.5	
RM7001–RM9000	1	0.8	
>RM9000	2	1.5	

RM: Malaysia Ringgit; RM100 approximately equivalent to USD25.

**Table 5:** Energy intake of males and females (*n* = 133).

Variable	Mean ± SD		*t*	*p*-Value
	Males (*n* = 54)	Females (*n* = 79)		
Total energy intake (kcal)	1741 ± 339	1625 247	2.165	0.033 *

* Variables differed significantly among groups as assessed by independent-sample *t*-test, *p* < 0.05.

**Table 6:** Difference between age and energy intake among respondents (*n* = 133).

Variable	Mean ± SD (kcal)	*F*	*p*-Value
Age (years)		4.244	0.016 *
<30 (*n* = 25)	1717 ± 201		
30–39 (*n* = 67)	1721 ± 263		
≥40 (*n* = 41)	1564 ± 355		

* Variables differed significantly among groups as assessed by ANOVA, *p* < 0.05.

**Table 7:** Post hoc tests for age groups

Age (Years)	Age (Years)	*p*-Value
<30	30–39	0.998
	≥40	0.090
30–39	<30	0.998
	≥40	0.017 *

* Variables differed significantly at *p* < 0.05.

**Table 8:** Preferences of food portion size based on plate size (*n* = 133).

Foods	Percentage (*%*)		*t*	*p*-Value
	23 cm Plate	28 cm Plate		
**Carbohydrate food (white rice, boiled)**				
1 scoop	34.6	15.8	4.390	<0.001 *
2 scoops	57.9	44.4	1.847	0.067
3 scoops	7.5	39.8	−7.277	<0.001 *
**Protein food (fried chicken)**				
1 small-sized	52.6	17.3	6.626	<0.001 *
1 moderate-sized	42.1	56.4	−2.422	0.017 *
1 large-sized	5.3	26.3	−5.464	<0.001 *
**Vegetable food (stir-fried water spinach)**				
½ scoop	48.9	9.8	4.869	<0.001 *
1 scoop	39.1	60.9	−3.538	0.001 *
1 ½ scoops	12.0	29.3	−4.175	<0.001 *

* Variables differed significantly among groups as assessed by paired-sample *t*-test, *p* < 0.05.

**Table 9:** Comparison of meal energy based on plate size between genders (*n* = 133).

Food Type	Meal Energy Mean ± SD (kcal)		*t*	*p*-Value
	23-cm Plate	28-cm Plate		
**Carbohydrate food (white rice, boiled)**				
All subjects (*n* = 133)	173 ± 59	224 ± 71	−9.464	<0.001 *
Male (*n* = 54)	181 ± 59	231 ± 82	−5.518	<0.001 *
Female (*n* = 79)	167 ± 59	219 ± 68	−7.738	<0.001 *
**Protein food (fried chicken)**				
All subjects (*n* = 133)	205 ± 81	282 ± 89	−10.442	<0.001 *
Male (*n* = 54)	210 ± 82	295 ± 95	−6.792	<0.001 *
Female (*n* = 79)	202 ± 81	273 ± 84	−7.934	<0.001 *
**Vegetable food (stir-fried water spinach)**				
All subjects (*n* = 133)	41 ± 17	55 ± 15	−10.706	<0.001 *
Male (*n* = 54)	38 ± 15	53 ± 17	−7.266	<0.001 *
Female (*n* = 79)	42 ± 18	56 ± 13	−7.854	<0.001 *
**Total meal energy**				
All subjects (*n* = 133)	419 ± 124	561 ± 143	−12.069	<0.001 *
Male (*n* = 54)	429 ± 127	579 ± 163	−7.240	<0.001 *
Female (*n* = 79)	411 ± 122	548 ± 127	−9.876	<0.001 *

* Variables differed significantly among groups as assessed by paired-samples *t*-test, *p* < 0.001.

**Table 10:** Differences between foodscape (colour) preference based on food type (*n* = 133).

Foods	Percentage (%)		*t*	*p*-Value
	Unicoloured	Multicoloured		
Rice	40.6	59.4	−2.021	0.001 *
Protein	27.8	72.2	−10.981	<0.001 *
Vegetable	34.6	65.4	−8.909	<0.001 *

* Variables differed significantly among groups as assessed by paired-samples *t*-test, *p* < 0.05.

**Table 11:** Comparison of meal energy of food selection by subjects between unicoloured and multicoloured food (*n* = 133)

Foods	Meal Energy Mean ± SD (kcal)	*t*	*p*-Value
**Rice**			
Unicoloured (White rice, boiled)(*n* = 54)	123 ± 43	−9.182	<0.001 *
Multicoloured (*Briyani rice*) (*n* = 79)	189 ± 37		
**Protein**			
Unicoloured (“*Paprika chicken*”) (*n* = 37)	123 ± 35	−5.367	<0.001 *
Multicoloured (“*Turmeric chicken*”) (*n* = 96)	163 ± 39		
**Vegetable**			
Unicoloured (Stir-fried water spinach) (*n* = 37)	51 ± 19	−15.763	<0.001 *
Multicoloured (“*Lodeh*”) (*n* = 96)	132 ± 32		

* Variables differed significantly among groups as assessed by independent-sample *t*-test, *p* < 0.001.

**Table 12:** Differences between foodscapes (shape) preference based on food type (*n* = 133).

Foods	Percentage (%)		*t*	*p*-Value
	Round	Cube		
Porridge	83.5	16.5		<0.001 *
Protein	49.6	50.4		0.754
Vegetable	77.4	22.6		<0.001 *

* Variables differed significantly among groups as assessed by paired-sample *t*-test, *p* < 0.001.

**Table 13:** Comparison of meal energy of food selection by subjects between round- and cube- shaped food (*n* = 133)

Foods	Meal Energy Mean ± SD (kcal)	*t*	*p*-Value
**Porridge**			
Round (Chicken porridge with round carrot) (*n* = 111)	64 ± 20	0.400	0.693
Cube (Chicken porridge with cubed carrot) (*n* = 22)	61 ± 29		
**Protein**			
Round (Fried round tofu) (*n* = 66)	93 ± 38	−3.025	0.03 *
Cube (Fried cubed tofu) (*n* = 67)	113 ± 38		
**Vegetable**			
Round (Stir-fried cabbage with round carrot) (*n* = 103)	51 ± 19	0.211	0.834
Cube (Stir-fried cabbage with cubed carrot) (*n* = 30)	50 ± 15		

* Variables differed significantly among groups as assessed by independent-samples *t*-test, *p* < 0.001.

**Table 14:** Association between foodscapes (colour and shape) and total meal energy.

Foodscapes	Meal Energy (kcal) *n* (%)		χ^2^	*p*-Value
	<1500	≥1500		
**Colour**				
**Rice**			9.017	0.003 *
Unicoloured	25 (61.0)	29 (31.5)		
Multicoloured	16 (39.0)	63 (68.5)		
**Protein**			4.556	0.033 *
Unicoloured	17 (41.5)	20 (21.7)		
Multicoloured	24 (58.5)	72 (78.3)		
**Vegetables**			10.787	0.001 *
Unicoloured	23 (56.1)	23 (25.0)		
Multicoloured	18 (43.9)	69 (75.0)		
**Shape**				
**Porridge**			0.754	0.385
Round carrot	32 (78.0)	79 (85.9)		
Cubed carrot	9 (22.0)	13 (14.1)		
**Protein**			0.481	0.488
Fried round tofu	18 (43.9)	48 (52.2)		
Fried cubed tofu	23 (56.1)	44 (47.8)		
**Vegetable**			7.889	0.005 *
Round carrot	25 (61.0)	78 (84.8)		
Cubed carrot	16 (39.0)	14 (15.2)		

* Variables differed significantly among groups as assessed by Chi-square test, *p* < 0.05.
